# Efficacy of Walking Adaptability Training on Walking Capacity in Ambulatory People With Motor Incomplete Spinal Cord Injury: A Multicenter Pragmatic Randomized Controlled Trial

**DOI:** 10.1177/15459683241248088

**Published:** 2024-04-25

**Authors:** Eline Zwijgers, Rosanne B. van Dijsseldonk, Marije Vos-van der Hulst, Juha M. Hijmans, Alexander C. H. Geurts, Noël L. W. Keijsers

**Affiliations:** 1 Department of Rehabilitation, Radboud University Medical Center, Donders Institute for Brain, Cognition and Behaviour, Nijmegen, The Netherlands; 2 Department of Research, Sint Maartenskliniek, Nijmegen, The Netherlands; 3 Department of Rehabilitation, Sint Maartenskliniek, Nijmegen, The Netherlands; 4 Department of Rehabilitation Medicine, University of Groningen, University Medical Center Groningen, Groningen, The Netherlands; 5 Department of Sensorimotor Neuroscience, Radboud University, Donders Institute for Brain, Cognition and Behaviour, Nijmegen, The Netherlands

**Keywords:** spinal cord injury, gait training, walking adaptability, walking capacity, rehabilitation, treadmill training

## Abstract

**Background and Objective:**

Balance and walking capacity are often impaired in people with motor incomplete spinal cord injury (iSCI), frequently resulting in reduced functional ambulation and participation. This study aimed to assess the efficacy of walking adaptability training compared to similarly dosed conventional locomotor and strength training for improving walking capacity, functional ambulation, balance confidence, and participation in ambulatory people with iSCI.

**Methods:**

We conducted a 2-center, parallel-group, pragmatic randomized controlled trial. Forty-one people with iSCI were randomized to 6 weeks of (i) walking adaptability training (11 hours of Gait Real-time Analysis Interactive Lab (GRAIL) training—a treadmill in a virtual reality environment) or (ii) conventional locomotor and strength training (11 hours of treadmill training and lower-body strength exercises). The primary measure of walking capacity was maximal walking speed, measured with an overground 2-minute walk test. Secondary outcome measures included the Spinal Cord Injury Functional Ambulation Profile (SCI-FAP), the Activities-specific Balance Confidence (ABC) scale, and the Utrecht Scale for Evaluation of Rehabilitation-Participation (USER-P).

**Results:**

No significant difference in maximal walking speed between the walking adaptability (n = 17) and conventional locomotor and strength (n = 18) training groups was found 6 weeks after training at follow-up (−0.05 m/s; 95% CI = −0.12-0.03). In addition, no significant group differences in secondary outcomes were found. However, independent of intervention, significant improvements over time were found for maximal walking speed, SCI-FAP, ABC, and USER-P restrictions scores. *Conclusions*. Our findings suggest that walking adaptability training may not be superior to conventional locomotor and strength training for improving walking capacity, functional ambulation, balance confidence, or participation in ambulatory people with iSCI.

**Trial Registration:**

Dutch Trial Register; Effect of GRAIL training in iSCI.

## Introduction

Although most people with motor incomplete spinal cord injury (iSCI) can walk after inpatient rehabilitation,^1^ the quality and efficiency of ambulation are affected.^2^ People with iSCI generally walk at a lower preferred speed^3^ and have an increased risk of falling.^4-6^ Furthermore, impaired walking capacity often restricts people with iSCI in the performance of mobility-related daily life activities (ie, functional ambulation)^7^ and their participation in the community (eg, work, household, and social activities).^8^ Hence, an important rehabilitation goal of people with iSCI is to improve their walking capacity to successfully ambulate in their home and community setting.^9^

Successful ambulation in daily life requires stepping, dynamic stability, and walking adaptability.^10^ Stepping is necessary to progress forward, while dynamic stability ensures an upright body position in space through control of the center of mass with respect to the changing base of support. Walking adaptability is the ability to modify the gait pattern when environmental circumstances change, encompassing both proactive and reactive gait adaptations.^11^ Proactive adaptations, triggered by visual stimuli, entail activities such as walking on irregular terrain or navigating through crowded areas, while reactive adaptations involve responses to mechanical perturbations, such as stumbling over a doorstep or withstand a strong crosswind. Currently, iSCI outpatient rehabilitation interventions mainly focus on the aspect of stepping to improve walking capacity.^12,13^ Examples of such locomotor interventions are overground or treadmill-based gait training with or without body-weight-support, as well as robotic-assisted gait training; either with or without manual assistance and/or functional electrical stimulation.^12,13^ In clinical practice, these interventions are usually combined with lower-body strength exercises to enhance muscle strength and overall effectiveness of the gait training. All these types of interventions show some potential for improving walking speed, but without established supremacy over another.^12,13^

In recent years, walking adaptability training has emerged as a potential rehabilitation intervention for people with iSCI, as it is expected to enhance walking capacity beyond the benefits of conventional interventions.^14,15^ Walking adaptability training targets the improvement of walking capacity by provoking gait adaptations through precision stepping, obstacle avoidance, and/or reacting to perturbations.^15-18^ In the work of van Dijsseldonk et al,^15^ ambulatory people with iSCI improved walking speed, dynamic stability, and balance confidence after 6 weeks of walking adaptability training in a virtual environment. This training increased walking speed by a similar amount as reported in the literature for conventional interventions.^19,20^ However, the number of training sessions of these conventional interventions was more than 3 times higher compared to the walking adaptability training. In a randomized controlled trial conducted by Yang et al,^14^ walking adaptability training (consisting of overground walking over obstacles and targets) was compared with treadmill-based gait training. This study reported significantly more improvement in endurance after treadmill-based gait training, along with similar improvements in walking speed, functional ambulation, and balance confidence for both interventions. However, it should be noted that the interventions were not directly comparable in terms of the number of steps per session, favoring treadmill-based gait training.^14^ Previous research has indicated that a higher dosage (ie, the number of trainings sessions as well as the number of steps per session) can positively influence the training effect.^21^ Hence, a study is needed to determine the efficacy of walking adaptability training versus conventional training for improving walking capacity, ensuring comparability in dosage between the interventions.

The primary objective of this pragmatic randomized controlled trial was to assess the efficacy of walking adaptability training compared to similarly dosed conventional locomotor and strength training for improving walking capacity in ambulatory people with iSCI. Walking capacity was operationalized as walking speed due to its established correlation with various functional ambulation skills, such as walking around curves, avoiding obstacles, and performing dual tasks.^22^ We hypothesized that walking adaptability training would improve walking speed more than conventional locomotor and strength training.^14,15,19,20^ In addition, we measured the effects of both interventions on secondary outcome measures, including functional ambulation, balance confidence, and participation in the community.

## Methods

### Study Design

A 2-center, parallel group, pragmatic randomized controlled trial was conducted at the Sint Maartenskliniek (SMK) and the University Medical Center Groningen (UMCG) in the Netherlands (trial register identifier: Dutch Trial Register; Effect of GRAIL training in iSCI). To ensure that both groups could benefit from the alternative training approach as well, the initial group comparison (including a follow-up period without intervention) was extended with a crossover design. The current article specifically focuses on the initial randomized controlled trial part of the overall study design. The study was approved by the regional medical ethics committee Oost-Nederland (NL69379.091.19) and by the internal review board of the Sint Maartenskliniek. All research activities were carried out in accordance with the guidelines and regulations of the Medical Research involving Human Subjects Act (WMO) and the Netherlands Code of Conduct for Research Integrity. Furthermore, the study was reported according to the consolidated standards of reporting trials.

### Participants

People with iSCI were recruited by their rehabilitation physician during visits to the outpatient clinic, including check-up appointments or self-initiated appointments to discuss complaints regarding their walking capacity. The following inclusion criteria were used: (1) being diagnosed with motor iSCI from a traumatic or non-traumatic origin (American spinal injury association Impairment Scale (AIS) C or D), (2) minimally 6 months post injury, (3) ability to walk at least 10 m with or without a walking aid, but without physical assistance, (4) ability to walk at comfortable speed between 0.3 and 1.0 m/s, (5) having a rehabilitation goal to improve walking capacity, (6) willingness and ability to cancel other interventions (eg, physiotherapy and botulinum toxin injections in the leg muscles) aimed at improving walking capacity during the study period, and (7) age ≥18 years. Exclusion criteria were: (1) other impairments of the nervous system or lower limbs that might affect walking or balance, (2) expected interference with one’s activity level by planned events such as an operation or moving, (3) walking adaptability training within the previous 6 months, and (4) insufficient understanding or mastery of the Dutch language. Participant characteristics (demographic, injury-related, and mobility-related) were registered at baseline. Demographic characteristics included age, weight, height, and sex. Injury-related characteristics included AIS, level of injury, time post injury, and cause. Furthermore, mobility-related characteristics included the functional ambulation categories and the use of walking aids outdoors and/or ankle foot orthoses.

### Procedures

Participants were randomly assigned to receive either walking adaptability or conventional locomotor and strength training in a 1:1 allocation ratio. The randomization process was performed using a computer-generated randomization schedule deployed in MATLAB (R2019b, MathWorks). To ensure balanced group assignments, the schedule employed permuted blocks of varying size (4 and 6). Group allocation was revealed by the MATLAB program only after participant enrollment. The investigators enrolled and assigned participants to their respective interventions. Participants, physiotherapists, and investigators were not blinded to group allocation due to the nature of the intervention. In addition, assessors were not blinded due to practical and organizational constraints. The assessment of the primary outcome measure occurred at 3 time points: baseline, immediately post-intervention, and at follow-up. The follow-up assessment took place at 6 weeks post-intervention and served as our primary endpoint. The assessment of secondary outcome measures was conducted at baseline and during the follow-up assessment.

### Interventions

Both interventions consisted of 11 training sessions of 60 minutes over a period of 6 weeks (on average 2 training sessions per week). The training interventions were designed to contain approximately 20 minutes of active walking to ensure a similar number of steps per session for both interventions. We chose a duration of 20 minutes based on clinical experience, as 20 minutes of walking is physically demanding for most iSCI individuals with limited walking capacity. The number of steps taken during a session were monitored with a pedometer (Polar A360; Polar Electro (SMK) and Fitbit Zip; Fitbit, Inc. (UMCG)). The level of physical tiredness before and after each training session was assessed using a 15-point scale ranging from 6 to 20 (similar to the Borg Rating of Perceived Exertion scale), where 6 represented “not tired at all” and 20 indicated “fully tired.” The perceived intensity of each training session was quantified as the difference between the physical tiredness ratings recorded at the end and the beginning of the session. Adherence was determined by counting the number of sessions completed by each individual. Participants were allowed to make up for missed sessions with a maximum of 2 sessions, which occasionally extended the training period to 7 weeks. Participants’ experience was assessed with a visual analogue scale measuring the subjective satisfaction of the received intervention on a range from 0 to 10, with higher scores indicating more satisfaction. Participants completed the visual analogue scale directly after finishing the intervention.

#### Walking Adaptability Training

The walking adaptability training was conducted using the Gait Real-time Analysis Interactive Lab (GRAIL; Motek Medical B.V.). The GRAIL incorporates an instrumented split-belt treadmill with adjustable pitch and sway, an 10-camera motion capture system (Vicon Motion Systems), and a 180° semi-cylindrical screen for the projection of synchronized virtual reality environments. The walking adaptability training was conducted by a physiotherapist certified to work with the GRAIL. For safety reasons, participants wore a safety harness attached to a rail on the ceiling without body weight support. During a training session, multiple walking adaptability tasks were performed, including precision stepping, obstacle avoidance, and/or reacting to perturbations. Precision stepping involved precise and accurate foot placement. Obstacle avoidance required participants to effectively maneuver around or step over virtually projected obstacles. Reacting to perturbations involved exposing participants to unexpected disturbances, such as sudden surface pitch or sway. The physiotherapist selected the tasks based on the participant’s goals and gradually increased training complexity according to the participant’s abilities. Based on a prior study^15^ and clinical experience, we learned that participants typically are able to engage in about 20 minutes of walking adaptability tasks during a 60 minutes session. Therefore, the physiotherapists were instructed to provide approximately 20 minutes of walking adaptability tasks. In the remaining time available during the session, physiotherapists could incorporate standing balance tasks, including weight shifting and/or performing foot clearance exercises during standing. The duration of active time performing standing balance tasks typically ranged from 0 to 10 minutes.

#### Conventional Locomotor and Strength Training

The conventional locomotor and strength training consisted of treadmill training and lower-body strength exercises and was conducted by a physiotherapist. The therapists were instructed to provide approximately 20 minutes of treadmill training. The physiotherapist adjusted the treadmill settings and walking speed to each participant’s individual physical and walking capacity and the progress the participant made during the intervention. In the remaining time available during the session, lower-body strength exercises were performed including leg press, seated leg curl, hip abduction, and/or adduction. The physiotherapist selected the strength exercises based on the participant’s abilities, and resistance of the strength exercises was gradually increased according to the number of correctly executed repetitions based on van de Goolberg’s strength-training rehabilitation system (Kracht Revalidatie Systeem).^23^ The duration of active time performing strength exercises typically ranged from 10 to 20 minutes.

### Primary Outcome Measure

The primary outcome measure was maximal walking speed as measured with an overground 2-minute Walk Test (2mWT), which is a valid and reliable test to assess walking capacity in people with iSCI.^24^ Participants were instructed to walk as far as possible, but safely, over an 18 m course. An examiner accompanied each participant for safety reasons, walking behind the participant to allow her/him to set the pace. Walking aids were allowed and kept constant between all 2mWT assessments. Also short rest breaks were allowed, but without stopping the time.

### Secondary Outcome Measures

The Spinal Cord Injury Functional Ambulation Profile (SCI-FAP) was used as secondary outcome measure to evaluate functional ambulation.^25^ The SCI-FAP includes 7 functional walking tasks, such as overcoming obstacles, doors, and stairs. The score is based on the time and assistance needed to complete the tasks at a comfortable pace. Higher scores indicate lower functioning (more time or assistance needed to complete tasks) with a maximum score of 2100. The assistance needed by a participant to complete a specific task was kept constant between all SCI-FAP assessments. This approach was chosen based on previous research demonstrating a correlation between SCI-FAP time and overall score changes.^26^ Moreover, this approach was adopted to prevent participants from modifying their required assistance at follow-up assessment, as they were aware of being scored on the assistance needed.

Balance confidence was measured with the Activities-specific Balance Confidence (ABC) scale.^27^ This scale comprises 16 items regarding different daily life activities. The total score ranges from 0 to 100, with higher scores indicating more balance confidence.

Participation was measured with the Utrecht Scale for Evaluation of Rehabilitation-Participation (USER-P).^28^ This scale comprises 31 items and covers 3 aspects of participation with 3 separate scales: frequency, restrictions, and satisfaction. Each subscale ranges from 0 to 100, with higher scores indicating higher levels of participation (higher frequency, less restrictions, higher satisfaction).

### Sample Size

The required sample size was calculated for an analysis of covariance (ANCOVA).^29^ This approach takes into account the correlation (*r*) between baseline and follow-up scores at the end of the treatment period, and it has been established that ANCOVA with n × (1 − *r*^2^) subjects in each group provides the same statistical power as a *t*-test with n subjects in each group based on a conventional power calculation. To estimate the expected effect of the walking adaptability training, we refer to the study by van Dijsseldonk et al.^15^ Within their dataset, we selected participants who met our current inclusion criteria (n = 9) and observed an increase in walking speed of 0.23 m/s. For the expected effect of the conventional locomotor and strength training, we examined 2 similar interventions among individuals with iSCI: treadmill-based gait training^14^ and resistance training combined with aerobic training.^19^ These interventions led to improvements in walking speed of 0.07 and 0.13 m/s, respectively, with a mean improvement of 0.10 m/s. Consequently, we expected a mean difference of 0.13 m/s (0.23 − 0.10 m/s) between walking adaptability training and conventional locomotor and strength training. To estimate the expected standard deviation (SD) in walking speed and the correlation between baseline and follow-up scores at the end of the treatment period, we refer to the study by van Dijsseldonk et al.^15^ Considering a statistical power of 80%, a 2-sided significance level of 5%, a correlation of 0.94, and a dropout rate of 10%, we determined that our study would require an inclusion of 40 participants with iSCI (20 in each group) to detect a mean group difference of 0.13 m/s (SD = 0.4 m/s) in walking speed.

### Statistical Analysis

All outcome measures at follow-up were compared between the group that received walking adaptability training and the group that received conventional locomotor and strength training using ANCOVA. The performance at baseline was included as a covariate. When the assumption of normality was violated, data transformations were performed. No intention-to-treat approach was followed, as we were primarily interested in the functional effects that could truly be attributed to the interventions.

The effect of time on maximal walking speed was analyzed using repeated-measures ANOVA (baseline, post-intervention, and follow-up) supplemented with post-hoc *t*-tests using Bonferroni correction. The effect of time on secondary outcome measures was analyzed using dependent *t*-tests. Data of both interventions were pooled if no significant group difference at follow-up was found.

The mean number of steps taken during a session, the perceived intensity, and participants’ experience were compared between groups using independent *t*-tests, or with Mann–Whitney *U* tests if the assumption of normality was violated.

All analyses were performed in SPSS version 25 (IBM Corp). The level of significance (α) was set at .05.

## Results

**Figure 1.**
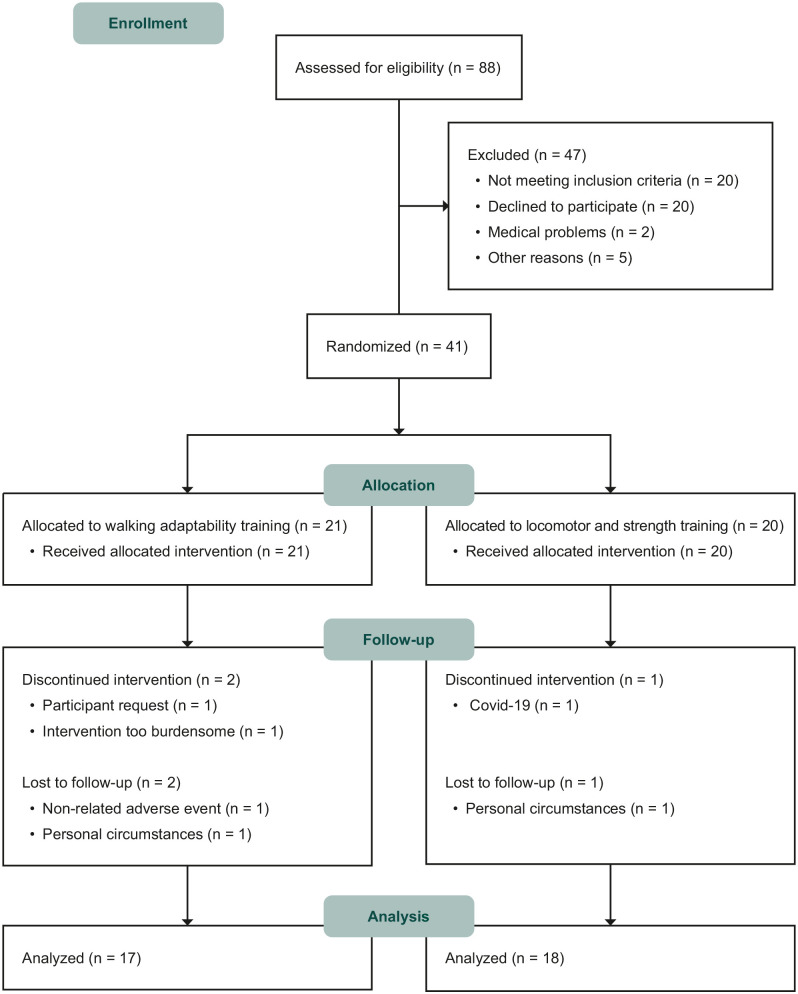
Flow diagram of participants.

Participant enrolment commenced August 2019, and the last participant completed follow-up in August 2022. In total, 41 participants were included (31 in the SMK and 10 in the UMCG), of whom 21 were allocated to the walking adaptability training group and 20 to the conventional locomotor and strength training group (see Figure 1). Thirty-five participants completed post-intervention and follow-up assessments and were included in the analysis. Participant characteristics at baseline are shown in Table 1.

**Table 1.** Participant Characteristics at Baseline.^a^

	Walking adaptability training group	Conventional locomotor and strength training group
N	17	18
Demographic		
Age (y)^b^	62 (56-71)	67 (60-72)
Weight (kg)^c^	84 ± 16	79 ± 13
Height (cm)^c^	176 ± 11	173 ± 11
Sex		
Men	10	9
Women	7	9
Injury-related		
AIS		
Grade C	2	1
Grade D	15	17
Level of injury		
Cervical	8	10
Thoracic	3	5
Lumbar	6	3
Time post injury (mo)^b^	47 (20-120)	66 (20-135)
Cause		
Traumatic	6	9
Non-traumatic	11	9
Mobility-related		
Functional ambulation categories		
Cat 4	4	4
Cat 5	13	14
Use of walking aids outdoors	12	13
Single-point cane or crutch	5	5
2 crutches	1	3
Walker	6	5
Use of ankle-foot orthoses	2	5

Abbreviation: AIS, American spinal injury association Impairment Scale.

a Values are reported as number of participants unless stated otherwise.

b Reported as median (interquartile range).

c Reported as mean ± standard deviation.

### Participants’ Adherence and Experience

All participants attended 9 or more training sessions of 60 minutes each, with the same median of 11 (range 9-11) sessions for both the walking adaptability and the conventional locomotor and strength training group. The number of steps per training session was somewhat higher for the walking adaptability training group (median (interquartile range (IQR)) = 2670 (2261-3352)) compared to the conventional locomotor and strength training group (median (IQR) = 2400 (1490-2555); *z* = −2.18, *P* = 0.03). The walking adaptability training group reported an average physical tiredness level of 9.0 (SD = 1.6) before the training session, which increased to 14.3 (SD = 1.6) after the session. The conventional locomotor and strength training group reported an average of 8.5 (SD = 2.2) before and 12.3 (SD = 2.0) after the training session. Thus, the perceived intensity, defined as the difference between the ratings of physical tiredness before and after, was higher for the walking adaptability training group (mean ± SD = 5.3 ± 1.9) compared to the conventional locomotor and strength training group (mean ± SD = 3.8 ± 2.0; *t*(33) = 2.36, *P* = .03). One adverse event (foot pain) during the walking adaptability training was reported, but did not lead to discontinuation of the intervention. Participant experience was not different between both groups with a median of 8.5 (range 7-10) for the walking adaptability training group and 9 (range 6-10) for the conventional locomotor and strength training group (*z* = −0.45, *P* = .67).

### Walking Capacity

**Figure 2.**
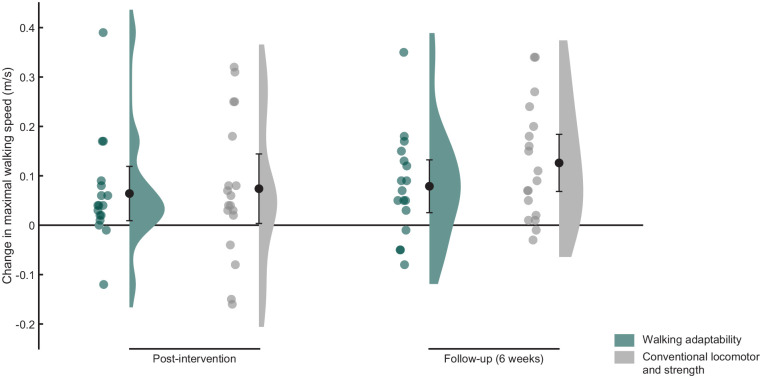
Raincloud plot of the changes in maximal walking speed with respect to baseline. Dots represent the individual data points and bars the means with 95% confidence intervals.

Maximal walking speed data at baseline, post-intervention, and follow-up are shown in Table 2 and Figure 2. ANCOVA showed no significant group difference in maximal walking speed at follow-up (*F*(1, 32) = 1.48, *P* = 0.23). The mean group difference in maximal walking speed at follow-up adjusted for group differences at baseline (adaptability—locomotor and strength training group) was −0.05 m/s (95% CI = −0.12 to 0.03).

**Table 2.** Walking Capacity at Baseline, Post-Intervention, and Follow-Up Assessment.^a^

Outcome	Walking adaptability training group (n = 17)			Conventional locomotor and strength training group (n = 18)			ANCOVA (mean group difference)^b^	ANCOVA (stats)
	Baseline	Post-intervention	Follow-up	Baseline	Post-intervention	Follow-up	Follow-up	Follow-up
Maximal walking speed (m/s)	0.81 ± 0.30	0.88 ± 0.31	0.89 ± 0.33	0.84 ± 0.28	0.91 ± 0.35	0.97 ± 0.33	−0.05 (−0.12-0.03)	*F*(1, 32) = 1.48, *P* = .23

Abbreviation: ANCOVA, analysis of covariance.

a Values are reported as mean ± standard deviation unless stated otherwise.

b Reported as mean (95% confidence interval); mean group difference in walking adaptability training—conventional locomotor and strength training, adjusted for group differences at baseline.

Repeated-measures ANOVA showed an effect of time (*F*(1.66, 56.36) = 16.87, *P* < .01). Post-hoc analysis showed significant improvement in maximal walking speed between baseline and post-intervention (*P* < .01) and between baseline and follow-up (*P* < .01). Independent of intervention, maximal walking speed increased by 0.07 m/s (95% CI = 0.03-0.11) at post-intervention and by 0.10 m/s (95% CI = 0.06-0.14) at follow-up relative to baseline.

### Functional Ambulation, Balance Confidence, and Participation

**Figure 3.**
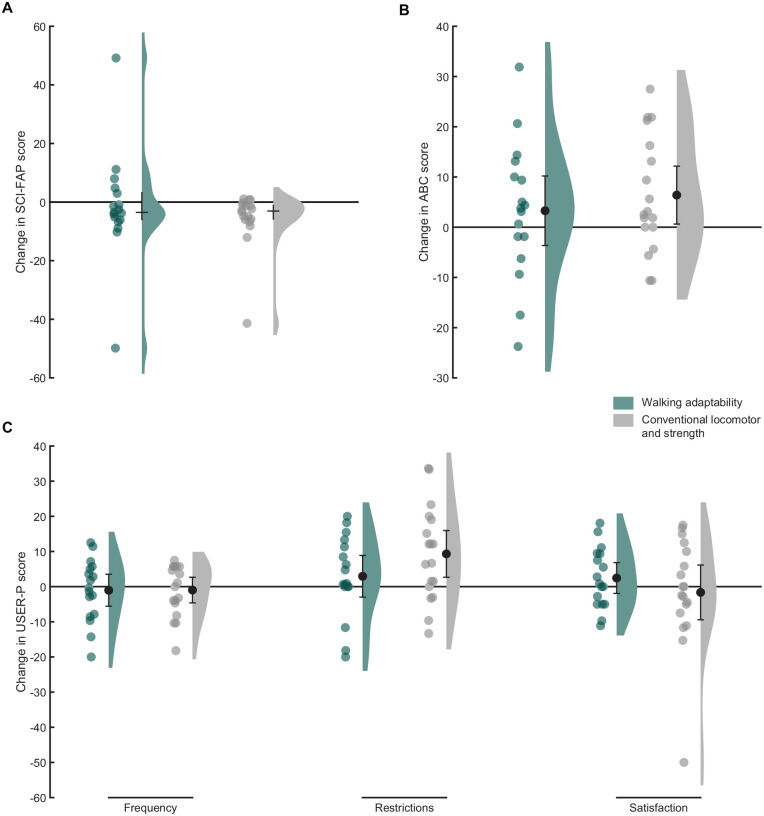
Raincloud plots of the changes in Spinal Cord Injury Functional Ambulation Profile (SCI-FAP) scores (A), the changes in Activities-specific Balance Confidence (ABC) scores (B), and the changes in Utrecht Scale for Evaluation of Rehabilitation-Participation (USER-P) scores (C) between baseline and follow-up. Dots represent the individual data points and bars the medians with interquartile ranges (A) or means with 95% confidence intervals (B and C).

ANCOVA showed no significant group differences in any secondary outcome measures at follow-up (Table 3; Figure 3). Independent of intervention, dependent *t*-tests revealed significant improvements across time between baseline and follow-up for the SCI-FAP score (median difference = −3.3 points, IQR = −6.0 to −0.3, *P* < .01), ABC score (mean difference = 4.9 points, 95% CI = 0.6-9.2, *P* = .03), and USER-P restrictions score (mean difference = 6.2 points, 95% CI = 1.8-10.6, *P* < .01).

**Table 3.** Functional Ambulation, Balance Confidence, and Participation at Baseline and Follow-Up.^a^

Outcome	Walking adaptability training group (n = 17)		Conventional locomotor and strength training group (n = 18)		ANCOVA (mean group difference)^b^	ANCOVA (stats)
	Baseline	Follow-up	Baseline	Follow-up	Follow-up	Follow-up
SCI-FAP score^c^	25 (15-49)	24 (10-49)	21 (13-38)	19 (11-34)	—^d^	*F*(1, 32) = 0.08, *P* = 0.79
ABC score	55 ± 18	59 ± 18	57 ± 19	64 ± 16	−3.7 (−11.5-4.1)	*F*(1, 32) = 0.94, *P* = .34
USER-P score						
Frequency	30 ± 8	29.12 ± 7.64	33 ± 8	32 ± 8	−1.7 (−6.7-3.3)	*F*(1, 32) = 0.48, *P* = .49
Restrictions	73 ± 17	75.75 ± 12.52	70 ± 17	79 ± 11	−5.0 (−11.1-1.2)	*F*(1, 32) = 2.71, *P* = .11
Satisfaction	64 ± 13	66 ± 7.2	67 ± 16	66 ± 17	2.3 (−5.1-9.7)	*F*(1, 32) = 0.41, *P* = .53

Abbreviations: SCI-FAP, spinal cord injury functional ambulation profile (range 0-2100); ABC, activities specific balance (range 0-100); USER-P, utrecht scale for evaluation of rehabilitation-participation (range 0-100); ANCOVA, analysis of covariance.

a Values are reported as mean ± standard deviation unless stated otherwise.

b Reported as mean (95% confidence interval); mean group difference in walking adaptability training—conventional locomotor and strength training, adjusted for group differences at baseline.

c Reported as median (interquartile range).

d Data are LOG-transformed, therefore no mean difference is reported.

## Discussion

Contrary to our hypothesis, we did not observe a superior effect of walking adaptability training over conventional locomotor and strength training for improving walking capacity—operationalized as maximal walking speed during a 2mWT—in individuals with iSCI. When comparing 2 gait training interventions, it is essential to ensure that they involve a similar dose, as a higher dosage (ie, number of training sessions and number of steps per session) can positively influence the training effect.^21^ Therefore, both training interventions in our study were designed to ensure the same number of sessions and a similar number of steps per session. The number of sessions was the same for both interventions; however, we observed that the mean number of steps taken during the walking adaptability training was about 10% higher compared to the conventional locomotor and strength training. It is interesting to note that the randomized controlled trial conducted by Yang et al^14^ reported a much greater group difference in the number of steps taken. Specifically, the number of steps taken during walking adaptability training was 3 times lower than during the treadmill-based gait training (400 vs 1200 steps). Yet, despite this large difference, the treadmill-based gait training only showed a significantly superior effect on 1 out of 6 walking capacity measures. Therefore, it seems unlikely that the relatively small difference in the number of steps in the current study has influenced our results. In addition to the dosage, the intensity of an intervention is an important determinant of effectiveness.^21,30^ Although the average active time for the walking adaptability training was lower compared to the conventional locomotor and strength training, we found that the difference in perceived intensity was higher for the walking adaptability training than for the conventional locomotor and strength training. Given the higher perceived intensity for the walking adaptability training, a larger effect on maximal walking speed compared to the conventional locomotor and strength training would have been logical but, instead, no significant group difference was found. Thus, our findings suggest that we can reject our hypothesis that walking adaptability training as provided in the current study is superior to conventional locomotor and strength training for improving maximal walking speed in ambulatory people with iSCI. This conclusion aligns with recently published research comparing walking adaptability training to conventional training in other neurological populations.^31,32^

Compared to conventional locomotor and strength training, walking adaptability training yielded similar effects on functional ambulation, balance confidence, and participation. This pattern of results is understandable given the non-differential effects of the interventions on maximal walking speed. Moreover, in people with other chronic neurological conditions, similar (non-differential) effects have been reported, such as after stroke.^32^

The lack of observed differences between the 2 interventions in our study may be attributed to the incorporation of lower-body strength exercises within the conventional training. This decision was guided by the common clinical practice of combining locomotor interventions with lower-body strength exercises, aiming to improve muscle strength and overall effectiveness of the gait training. However, it is worth noting that despite including both treadmill training and lower-body strength exercises in the conventional training, the observed improvements in walking speed after the intervention fell within the range of previously reported changes following just treadmill training.^14,33^ Furthermore, a recent review by Hornby et al^34^ has highlighted inconsistent evidence regarding the potential benefits of lower-body strength training on walking speed. This suggests that the addition of lower-body strength exercises may not have yielded benefits beyond what is typically achieved with treadmill training alone. An alternative cause of the non-differential results observed in this study may be attributed to variations in participant motivation. Previous research has highlighted differences in the improvement of physical function during rehabilitation between highly motivated and less motivated stroke survivors.^35^ However, it is noteworthy that participant experiences were similar for both interventions, suggesting that motivation levels did not significantly influence the study outcomes.

It is important to recognize that, independent of intervention, maximal walking speed increased by 0.07 m/s after 6 weeks of training, which result was retained or even reinforced at 6-week follow-up with a 0.10 m/s improvement relative to baseline. The amount of improvement at follow-up is clinically relevant, as it aligns with the reported minimal clinically important difference (MCID) of 0.10 m/s for walking speed.^36^ Previous research on gait training in individuals with iSCI has revealed variable results concerning improvements in walking speed, ranging from minimal changes (around 0.00 m/s) to significant increases (up to 0.16 m/s).^14,19,20,33,37,38^ Our study’s findings on improvement in walking speed lie around the midpoint of this range, suggesting a noteworthy effect on participants’ walking speed compared to previous studies. Concurrently with the observed improvement in maximal walking speed in our study, both functional ambulation and balance confidence increased at follow-up compared to baseline, regardless of the intervention. Participants also reported less participation restrictions in daily life activities, such as work, household chores, and social activities. At an individual level, 15 out of 35 participants (43%) showed improvements in maximal walking speed that exceeded the MCID of 0.10 m/s at follow-up.^36^ No MCID values for the secondary outcome measures in the iSCI population have been reported in the existing literature. Therefore, we employed a distribution-based approach to determine the MCID for the SCI-FAP, ABC, and USER-P restrictions scores, which was 0.5 SD of their baseline values, according to a systematic review conducted by Norman et al.^39^ Similar to the observed improvement in maximal walking speed, 37% (13/35) of the participants exceeded the MCID on the ABC scale (9.1 points) and 43% (15/35) exceeded the MCID on the USER-P restrictions scale (8.5 points), indicating a clinically meaningful change. For the SCI-FAP score, only 6% (2/35) of the participants exceeded the MCID (21.9 points).

Since we found no evidence that walking adaptability training is superior to conventional locomotor and strength training, a rehabilitation professional may indicate both interventions for an individual with iSCI to enhance walking capacity and functional ambulation in the home and community setting. Based on participant experience, there does not seem to be an overall preference either. The conventional locomotor and strength training in our study implicated a treadmill and lower body exercise machines, which combination can be more easily integrated into the community care system compared to walking adaptability training using the GRAIL system. Therefore, we believe that conventional (locomotor and strength) training as provided in our study holds the greatest potential for widespread use.

Our study had some limitations that should be considered when interpreting the results. First, the sample size was calculated based on improvements in walking speed observed in previous studies that were not directly comparable to our specific study design in terms of the number of training sessions or timing and type of assessment. This may have introduced a source of error in our sample size calculation. Therefore, we cannot rule out the possibility that a significant group difference would have been observed with a larger sample size. Second, the study was partly conducted during the COVID-19 pandemic, which may have influenced the results of the USER-P frequency subscale, as participants were restricted in their ability to engage in leisure and social activities. Third, both the SCI-FAP and USER-P restrictions subscales have a ceiling effect,^25,28^ which may have reduced their responsiveness given the relatively high baseline performance of our participants. Fourth, it is important to note that the assessors were not blinded, which may have introduced bias into the assessment process. Fifth, a limitation of this study is the notion that the dosing of the interventions may have been suboptimal for certain participants. To ensure consistency, we selected a duration of 20 minutes of active walking based on clinical experience, aiming to achieve a comparable number of steps per session for both interventions. However, it is possible that some participants may have been capable of performing more than 20 minutes of active walking. Sixth, we did not evaluate the effectiveness and quality of the participants’ gait performance during the walking adaptability training. This limitation arises from the complexity inherent in assessing certain performance outcomes on the GRAIL system. While some outcomes can be quantified by a single parameter, such as completion time or the number of successful maneuvers, others involve multiple measures or less objective assessments, such as reactions to perturbations or an application combining precision stepping, obstacle avoidance and perturbations. Therefore, reporting an outcome to indicate the effectiveness and quality of the participants’ gait performance across all applications turned out to be unfeasible. Nevertheless, it is important to note that the physiotherapists have extensive experience in tailoring interventions to the capacities of individuals with iSCI, with the objective to adjust training sessions to the quality of participants’ gait performance. Finally, while the primary outcome walking speed has shown correlations with various functional ambulation skills,^22^ it may overlook or fail to fully capture the concept of walking adaptability. Unfortunately, at the start of our study, alternative outcome measures more closely related to walking adaptability were either unavailable or too much reliant on a certain level of walking proficiency.^26^ To address this limitation and better capture the concept of walking adaptability in future studies, it is crucial to develop and incorporate outcome measures more closely related to walking adaptability. One promising measure that could be considered is the Walk Ladder Test, recently developed by Kuijpers et al,^40,41^ as it specifically assesses walking adaptability. Implementing such measures in future studies would provide a more targeted evaluation of the efficacy and impact of walking adaptability interventions in people with iSCI.

## Conclusions

Our findings suggest that walking adaptability training may not be superior to conventional locomotor and strength training for improving walking capacity, functional ambulation, balance confidence, or participation in ambulatory people with iSCI. Yet, both interventions showed improvements on all outcome measures at 6 weeks after the intervention. These findings suggest that both walking adaptability training and conventional locomotor and strength training can be considered viable options for enhancing walking capacity and functional ambulation in the home and community setting in individuals with iSCI.
